# Selective protein degradation ensures cellular longevity

**DOI:** 10.7554/eLife.17185

**Published:** 2016-06-01

**Authors:** Sandra Malmgren Hill, Thomas Nyström

**Affiliations:** Institute of Biomedicine, Sahlgrenska Academy, University of Gothenburg, Göteborg, Sweden; Institute of Biomedicine, Sahlgrenska Academy, University of Gothenburg, Göteborg, Swedenthomas.nystrom@cmb.gu.se

**Keywords:** mitochondria, autophagy, lysosome, aging, quality control, *S. cerevisiae*

## Abstract

A previously unknown pathway can selectively degrade mitochondrial proteins in aged and stressed cells without destroying the organelle itself.

**Related research article** Hughes AL, Hughes CE, Henderson KA, Yazvenko N, Gottschling DE. 2016. Selective sorting and destruction of mitochondrial membrane proteins in aged yeast. *eLife* **5**:e13943. doi: 10.7554/eLife.13943

**Image** Yeast cells containing mitochondrial-derived compartments (green) attached to mitochondria (red)

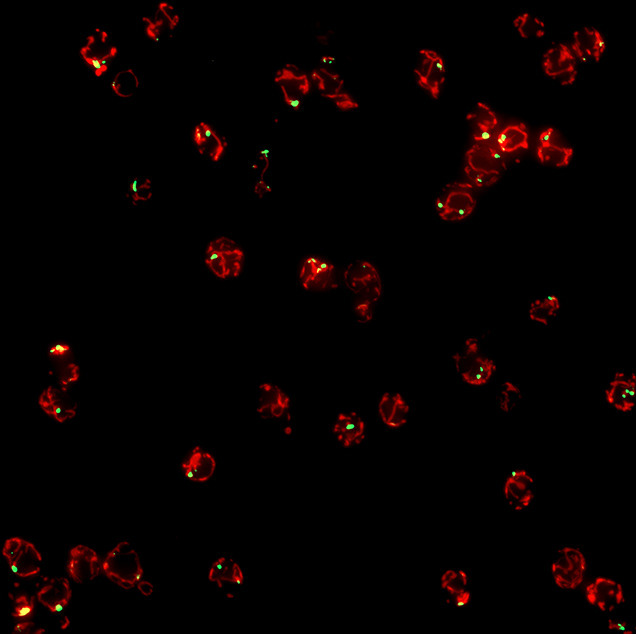

Mitochondria provide cells with energy and metabolite molecules that are essential for cell growth, and faulty mitochondria cause a number of severe genetic and age-related diseases, including Friedreich's ataxia, diabetes and cancer (Held and Houtkooper, 2015; Suliman and Piantadosi, 2016). To maintain mitochondria in a fully working state, cells have evolved a range of quality control systems for them (Held and Houtkooper, 2015). For example, faulty mitochondria can be removed through a process called mitophagy. In this process, which is similar to autophagy (the process used by cells to degrade unwanted proteins and organelles), the entire mitochondrion is enclosed by a double membrane. In yeast cells this structure fuses with a compartment called the vacuole, where various enzymes degrade and destroy the mitochondrion (Youle and Narendra, 2011). In animal cells an organelle called the lysosome takes the place of the vacuole.

Now, in eLife, Adam Hughes, Daniel Gottschling and colleagues at the Fred Hutchinson Cancer Research Center and University of Utah School of Medicine report evidence for a new quality control mechanism that helps to protect mitochondria from age- and stress-related damage in yeast (Hughes et al., 2016). In this mechanism, a mitochondrion can selectively remove part of its membrane to send the proteins embedded in this region to the lysosome/vacuole to be destroyed, while leaving the remainder of the mitochondrion intact (Figure 1).

**Figure 1.**
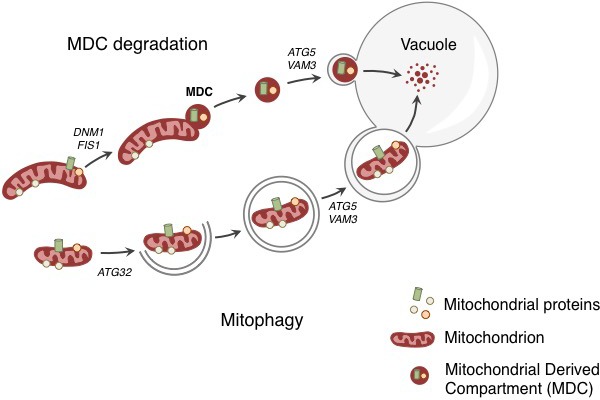
Degradation of mitochondria and selected mitochondrial membrane proteins. Yeast cells respond to stress and aging by sending a specific set of mitochondrial membrane proteins into the vacuole for degradation (top). This pathway involves the formation of a mitochondrial derived compartment (MDC), a process that is regulated by genes that are required for mitochondrial fission (*DNM1* and *FIS1*). The fusion of the MDC to the vacuole depends on genes that are involved in the late stages of autophagy and mitophagy (*ATG5* and *VAM3*). The MDC degradation pathway is distinct from the process of mitophagy (bottom), which involves the whole mitochondrion being enclosed in a membrane (grey lines) as a result of the activity of a gene called *ATG32*. The membrane-enclosed mitochondrion is then transported to the vacuole, where it is degraded.

Hughes et al. tracked the fate of Tom70, a protein that is found in the outer membrane of mitochondria, and discovered that it accumulated in the vacuole as the yeast aged. This accumulation was not the result of mitophagy, as Tom70 was directed to the vacuole even when a gene required for mitophagy was absent. Previous reports have linked cellular aging to a decline in mitochondrial activity (McMurray and Gottschling, 2003), which is caused by an earlier loss of pH control in the vacuole (Hughes and Gottschling, 2012). For this reason, Hughes et al. tested whether a drug that disrupts the pH of the vacuole triggers the degradation of Tom70. This appears to be the case – the drug caused Tom70 to move from the mitochondria to the vacuole for degradation.

Before Tom70 ended up in the vacuole it accumulated in a mitochondrial-derived compartment (MDC) at the surface of the mitochondria, close to the membrane of the vacuole. The formation of this compartment depended on the machinery that drives the process by which mitochondria divide. However, the subsequent delivery of the contents of the MDC to the vacuole used factors that are required for the late stages of autophagy (Figure 1).

Hughes et al. found that the MDC contained Tom70 and 25 other proteins, all of which are mitochondrial membrane proteins that rely on Tom70 to import them into the mitochondrial membrane. This suggests that the MDC degradation pathway selectively removes a specific group of mitochondrial proteins. An obvious question, therefore, is: how and why does disrupting the ability of the vacuole to control its pH trigger the selective delivery and degradation of these proteins?

Hughes et al. hypothesize that MDC formation is linked to metabolite imbalance, as the loss of acidity inside the vacuole prevents amino acids from being stored there (Klionsky et al., 1990). This in turn leads to a build up of amino acids in the cytoplasm that can overburden the transport proteins that import them into the mitochondria (Hughes and Gottschling, 2012). In this scenario, the selective degradation of mitochondrial transport proteins by the MDC pathway can be seen as a response that protects the organelle against an unregulated, and potentially harmful, influx of amino acids.

While MDCs still formed in very old yeast cells, they remained in vesicle-like structures in the cytoplasm and were not delivered to the vacuole. It therefore appears possible that the accumulation of undegradable cytosolic MDCs in the cytoplasm can contribute to the decline of the aging cell, much like the accumulation of protein aggregates does (Nystrom and Liu, 2014; Hill et al., 2014).

The discovery of MDCs and their role in delivering specific mitochondrial proteins to the vacuole for degradation raises a number of questions that may shed further light on both this process and the trials of aging. Do the MDCs contain regulatory factors that are required for delivering proteins into the vacuole? Why do MDCs fail to be delivered into the vacuole in old cells? The degradative enzymes inside the vacuole do not work well under conditions of elevated pH. It will therefore also be important to learn how long the degradation of mitochondrial membrane proteins can be maintained under these conditions (Kane, 2006).
